# The naturally occurring peptide GHK reverses age-related fibrosis by modulating myofibroblast function

**DOI:** 10.31491/apt.2024.12.158

**Published:** 2024-12-28

**Authors:** Qianpei He, Jordan Mazzola, Warren Ladiges

**Affiliations:** a Department of Comparative Medicine, School of Medicine, University of Washington, Seattle, WA 98195, USA.

**Keywords:** GHK peptide, fibroblasts, myofibroblasts, senescence, fibrosis, idiopathic pulmonary fibrosis

## Abstract

Fibrotic disorders, such as idiopathic pulmonary fibrosis, are characterized by the accumulation of myofibroblasts, cells responsible for excessive extracellular matrix deposition and tissue remodeling. The inability to terminate this reparative process leads to persistent fibrosis with increasing age. GHK (glycyl-L-histidyl-L-lysine], a naturally occurring peptide, has demonstrated the potential in modulating fibrotic pathways by reversing cellular senescence and inducing apoptosis in myofibroblasts. GHK promotes tissue regeneration and enhances wound healing by activating stemness markers like p63 and PCNA. In aging, GHK's effect on pulmonary fibroblasts may restore youthful phenotypes, improving fibroblast migration and collagen contraction. This commentary discusses the role of GHK in resolving persistent fibrosis and the molecular mechanisms underpinning these effects, including integrin-β1 signaling. The potential of GHK as a therapeutic agent for fibrosis, including combination strategies with antioxidants or anti-inflammatory agents, is also explored.

The loss of cellular homeostasis in fibrosis is characterized by the accumulation of activated fibroblasts, known as myofibroblasts [1], which are responsible for excessive extracellular matrix deposition and tissue remodeling. The inability to terminate this reparative process leads to persistent fibrosis with increasing age. Idiopathic pulmonary fibrosis (IPF) is a progressive and fatal interstitial lung disease affecting the elderly, reflecting the significant burden it places on the aging population [2]. Despite advances in the understanding of its clinical features, effective treatments remain elusive, necessitating further exploration of the underlying mechanisms and novel therapeutic targets.

The myofibroblast is a key effector cell in fibrotic disorders [3], driving extracellular matrix synthesis and tissue remodeling in progressive fibrosis [4]. Persistent activation and accumulation of myofibroblasts without resolution of the reparative response underlies the progressive nature of fibrotic reactions in injured tissue [5]. Autopsy studies of elderly patients with IPF have highlighted the severity and irreversibility of fibrotic remodeling in aged tissues, underscoring the importance of targeted therapeutic interventions [6].

Emerging evidence suggests that targeting senescence and associated pathways could offer novel therapeutic strategies for IPF. GHK (glycyl-L-histidyl-L-lysine) peptide has been shown to positively influence gene expression by regulating various cellular pathways associated with tissue repair, wound healing, and anti-aging mechanisms [7], and thus has potential as an anti-fibrotic agent. This peptide is naturally present in human plasma and is FDA approved for use in anti-aging skin creams. Its discovery originated from studies comparing human plasma from young and older adults, with the younger plasma being more effective in inducing macromolecular synthesis in rat hepatocytes and hepatoma cells [8, 9]. The active factor was found to be GHK, with human plasma levels around 200 ng/mL at 20 years of age, declining to less than 60 ng/mL at 60 years of age. Similar observations have been reported in animal models, including mice. Numerous studies have demonstrated that GHK accelerates wound healing and tissue regeneration, increases collagen contraction, and triggers the secretion of factors that promote mesenchymal cell activation [10-13]. It has been identified as a promising therapeutic candidate for fibrotic disorders due to its ability to decrease senescence and reverse apoptosis resistance in myofibroblasts [14, 15].

Choi *et al*. [12] reported that GHK increased the expression of p63 and PCNA, markers of stemness and cellular proliferation, in a keratinocyte model. p63 is a stem cell marker that belongs to a family that includes two structurally related proteins, p53 and p73 [16], while PCNA, which is present in proliferating cells throughout the cell cycle, is a well-established marker of proliferating cells [17]. These findings suggest that GHK may enhance the regenerative potential of various cell types by increasing their proliferative capacity. Our own research has shown that GHK promotes the migration of lung fibroblasts from aged mice (24 months old) in a dose-dependent manner (Figure 1), indicating that it can revert aged fibroblasts to a more youthful phenotype, potentially through the activation of mesenchymal progenitor cells. Our preliminary data also suggest that GHK increases the expression of p63 and PCNA in primary lung fibroblast cultures from aged mice.

Senescent and apoptosis-resistant lung fibroblasts have been shown to contribute to the persistence and progression of fibrosis [15]. A previous study from our lab showed that primary lung fibroblasts isolated from aged mice have a senescent phenotype [18]. In irradiated 3T3 fibroblasts, GHK treatment decreased the expression of p21, a key marker of cellular senescence (Figure 2A). Similarly, in fibroblasts from 26-month-old C57BL/6 mice, GHK reduced p21 and p53 expression (Figure 2B), further supporting its role in suppressing the senescent phenotype.

By suppressing senescence, GHK can facilitate the resolution of the persistent fibrotic process by enhancing the apoptotic removal of excess myofibroblasts. This process could shift the fibrotic response from pathological persistent collagen deposition to physiological collagen contraction. GHK has been shown to enhance extracellular matrix (ECM) activity in both dermal and pulmonary fibroblasts. The molecular systems involved include actin cytoskeletal remodeling and integrin signaling, with focal adhesion pathways facilitating attachment of fibroblasts to collagen. Integrin beta 1 (ITGβ1), a critical cell surface protein, is actively expressed in the presence of GHK and thus may serve as a primary target for its anti-fibrotic effect [14].

Recent evidence suggests that cellular senescence plays a role in promoting fibrosis, especially in aged tissues, where stress-induced senescent myofibroblasts may exhibit delayed clearance with age. Our research indicates that myofibroblast markers, such as alpha smooth muscle actin (α-SMA), are more prevalent in fibroblasts from aged mouse lungs compared to younger controls (Figure 3), highlighting the age-dependent increase in fibrosis susceptibility. GHK has also been shown to reverse radiation-induced senescence in the dermis by restoring its proliferative capacity [10].

Studies have highlighted the role of persistent fibrosis in aged tissues, where myofibroblasts in injured tissues of aged mice acquire a sustained senescent and apoptosis-resistant phenotype, exacerbating fibrosis (Hecker et al., 2014). GHK treatment reduces the IGF-dependent secretion of the fibrogenic growth factor (TGF-β1) [19], aiding in the resolution of persistent fibrosis and scarring. Using a collagen gel contraction assay, we observed that primary fibroblasts from the lungs of aged GHK-treated mice exhibited enhanced contraction compared to fibroblasts from placebo-treated mice (Figure 4), further supporting the hypothesis that GHK promotes the resolution of collagen scars.

Cellular senescence is closely linked to fibrosis, as demonstrated by nonproliferative, p16-positive myofibroblasts in fibrotic lung tissues [15]. Such observations have also been seen in human liver cirrhosis [20]. Resolution of fibrosis is preceded by apoptosis of myofibroblasts and clearance of the extracellular matrix [21]. Thus, the transition from “physiological” to “pathological” fibrosis is driven by impaired apoptosis of myofibroblasts, as well as excessive ECM deposition and accumulation, resembling a persistent wound-healing response. Senescent fibroblasts exhibit higher expressions of Bcl-2, conferring resistance to apoptosis [22]. Bcl-2 overexpression has been observed in fibroblasts isolated from aged mice with bleomycin-induced lung fibrosis [15], consistent with this mechanism. GHK appears to directly target myofibroblasts, converting pathological persistent collagen deposition to physiological collagen contraction through ITG-β1 activation and apoptotic elimination of excess myofibroblasts (Figure 5). In preclinical studies, complete resolution of fibrosis in preclinical studies would be the ideal outcome, advancing GHK towards clinical trials. Should fibrosis remain partially unresolved, alternative strategies could enhance the anti-fibrotic effects of GHK. One possibility would be combining GHK with antioxidants, such as the mitochondrial targeted antioxidant SS31 [23], given the role of redox imbalance in persistent lung fibrosis [15]. Another potential approach could involve combining GHK with an anti-inflammatory agent. While GHK has been shown to inhibit the inflammatory response and epithelial-mesenchymal transition (EMT) via the TGF-β1/Smad 2/3 and IGF-1 pathways in bleomycin-induced fibrosis models [24], its broader anti-inflammatory effects in the context of pulmonary fibrosis pathogenesis may warrant further exploration.

## Figures and Tables

**Figure 1.**
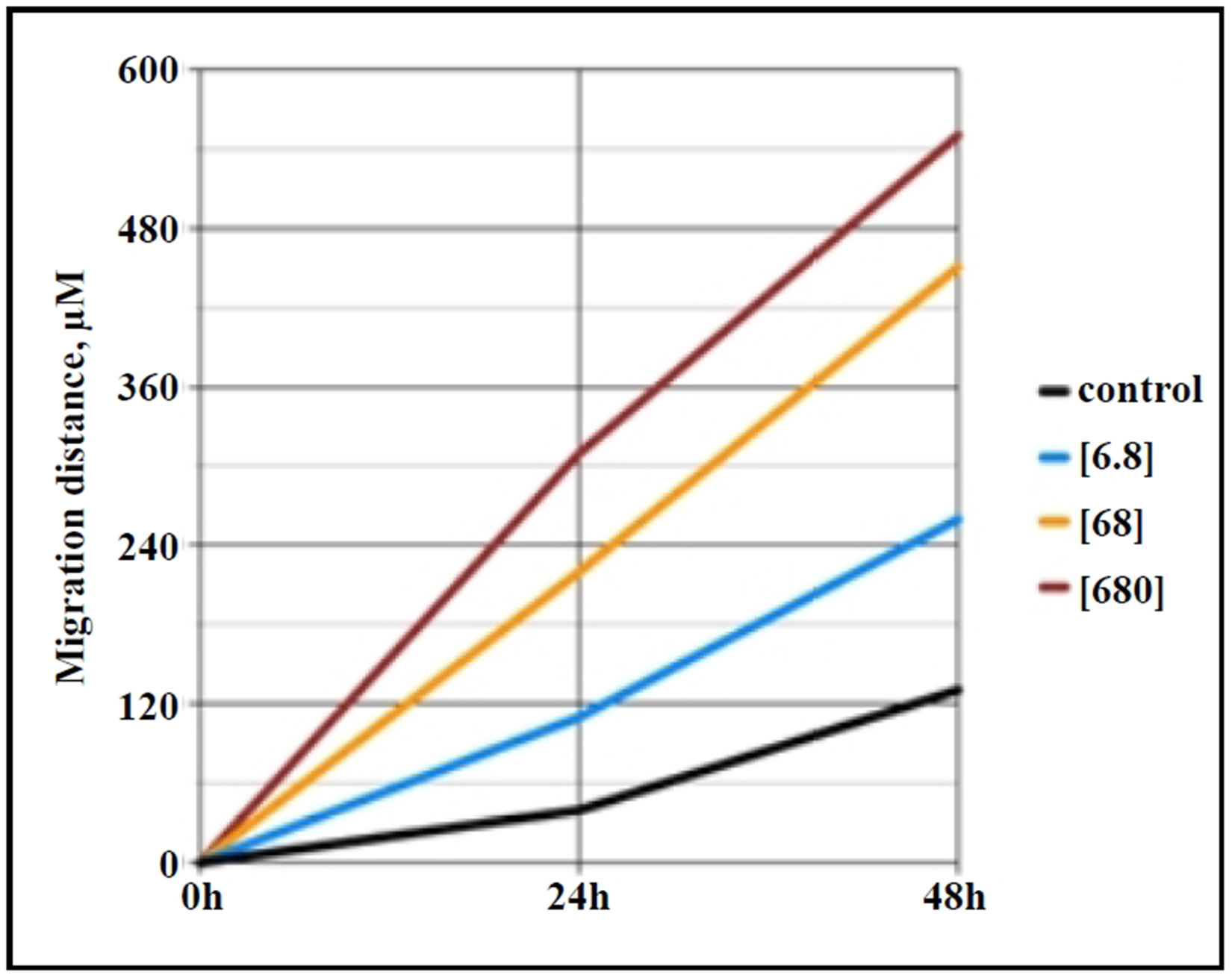
Migration distance (μm) across the gap of monolayer primary lung fibroblasts (*in vitro* scratch assay) from aged (24 months) C57BL/6 mice shows increased migration with rising GHK concentrations (ng/ mL) over 0-48 hours.

**Figure 2.**
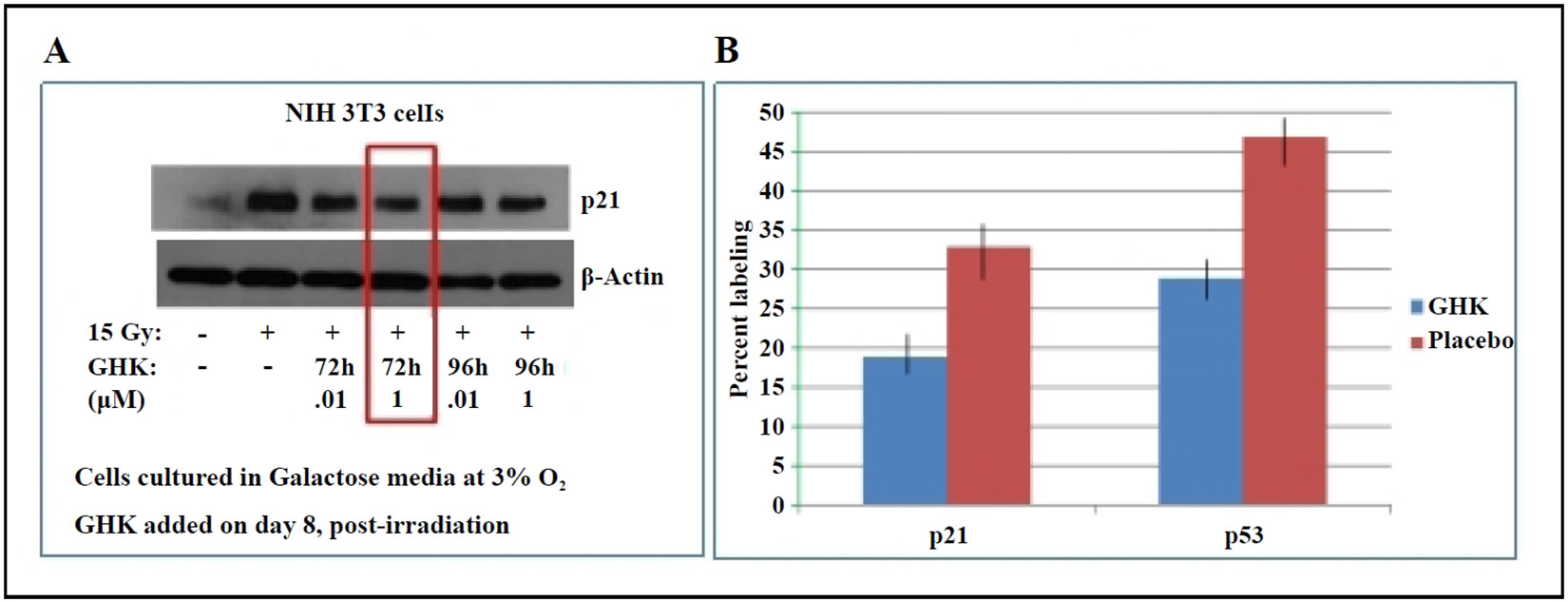
**(A)** GHK decreases p21 expression in irradiated NIH 3T3 fibroblasts. **(B)** GHK reduces p21 and p53 expression in primary lung fibroblasts from old (26 months) C57BL/6 mice, *P* ≤ 0.02.

**Figure 3.**
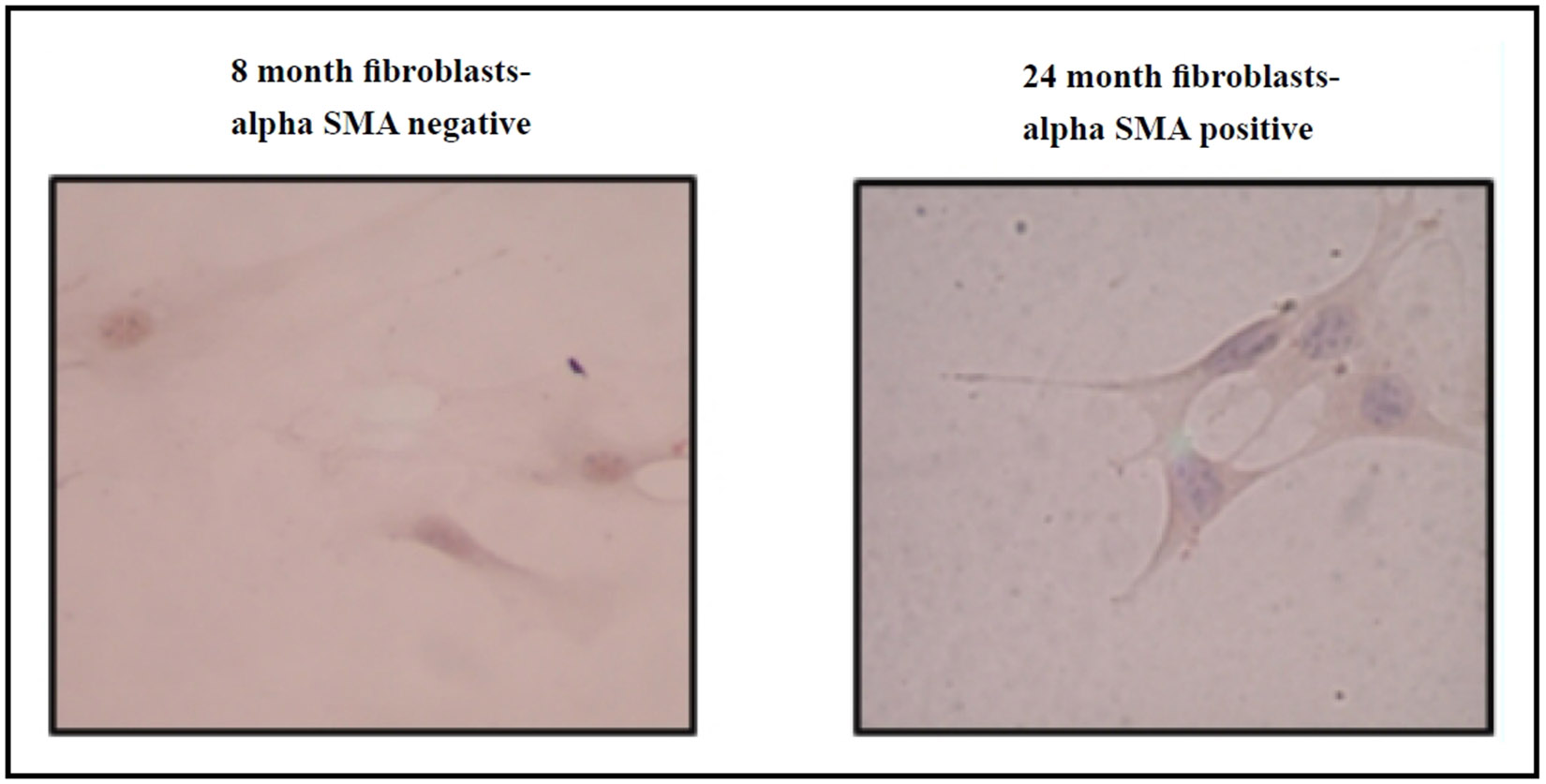
Increased α-SMA expression in primary lung fibroblasts from aged (24 months) C57BL/6 mice compared to young (8 months) C57BL/6 mice.

**Figure 4.**
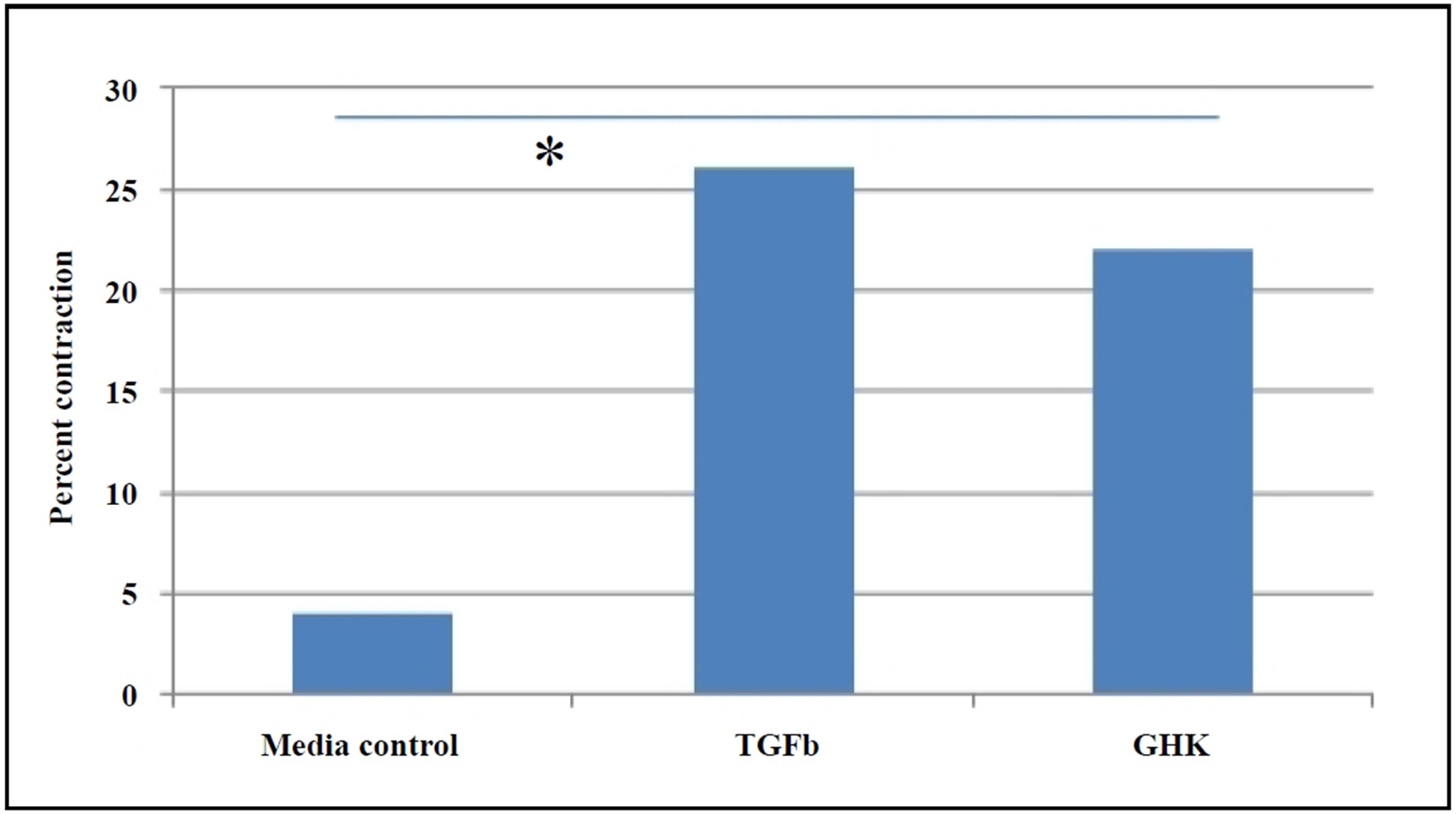
GHK enhances collagen gel contraction in primary lung fibroblasts from aged (26 months) C57BL/6 mice, which was determined by percent contraction from initial size, *P* ≤ 0.05.

**Figure 5.**
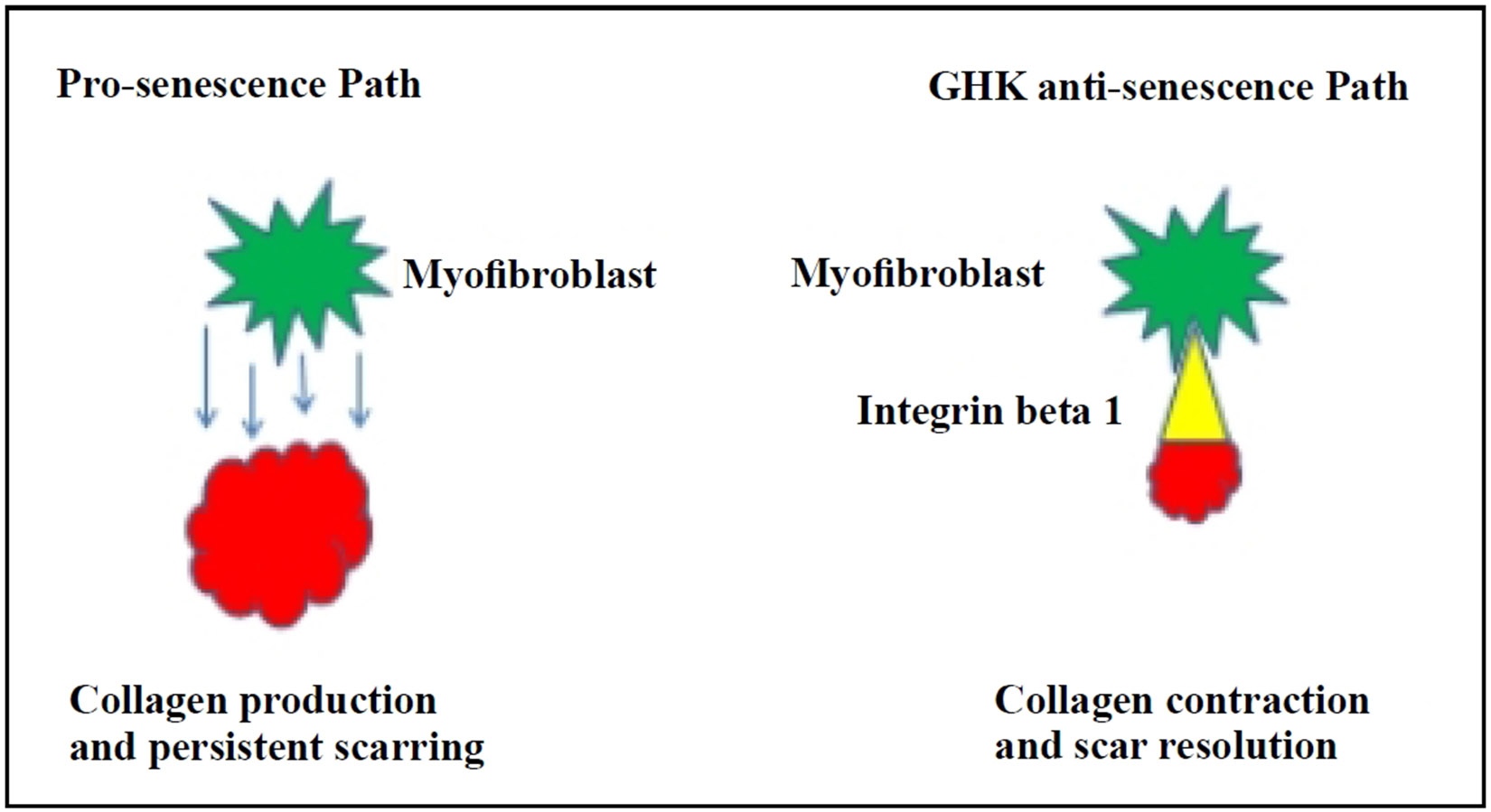
Proposed mechanism of GHK: reversal of senescence in lung fibroblasts and activation of integrin (ITG)-β1 to resolve persistent fibrosis.
